# Your Eyes Give You Away: Prestimulus Changes in Pupil Diameter Correlate with Poststimulus Task-Related EEG Dynamics

**DOI:** 10.1371/journal.pone.0091321

**Published:** 2014-03-11

**Authors:** Linbi Hong, Jennifer M. Walz, Paul Sajda

**Affiliations:** Department of Biomedical Engineering, Columbia University, New York, New York, United States of America; Centre de Neuroscience Cognitive, France

## Abstract

Pupillary measures have been linked to arousal and attention as well as activity in the brainstem's locus coeruleus norepinephrine (LC-NE) system. Similarly, there is evidence that evoked EEG responses, such as the P3, might have LC-NE activity as their basis. Since it is not feasible to record electrophysiological data directly from the LC in humans due to its location in the brainstem, an open question has been whether pupillary measures and EEG variability can be linked in a meaningful way to shed light on the nature of the LC-NE role in attention and arousal. We used an auditory oddball task with a data-driven approach to learn task-relevant projections of the EEG, for windows of data spanning the entire trial. We investigated linear and quadratic relationships between the evoked EEG along these projections and both prestimulus (baseline) and poststimulus (evoked dilation) pupil diameter measurements. We found that baseline pupil diameter correlates with early (175–200 ms) and late (350–400 ms) EEG component variability, suggesting a linear relationship between baseline (tonic) LC-NE activity and evoked EEG. We found no relationships between evoked EEG and evoked pupil dilation, which is often associated with evoked (phasic) LC activity. After regressing out reaction time (RT), the correlation between EEG variability and baseline pupil diameter remained, suggesting that such correlation is not explainable by RT variability. We also investigated the relationship between these pupil measures and prestimulus EEG alpha activity, which has been reported as a marker of attentional state, and found a negative linear relationship with evoked pupil dilation. In summary, our results demonstrate significant relationships between prestimulus and poststimulus neural and pupillary measures, and they provide further evidence for tight coupling between attentional state and evoked neural activity and for the role of cortical and subcortical networks underlying the process of target detection.

## Introduction

The locus coeruleus (LC) is a small nucleus located in the dorsal pons. The LC is known to have widespread ascending projections throughout the brain, and is the main source for cortical norepinephrine (NE) [1], [2]. Traditional studies have linked the LC-NE system with arousal and attention [3]. Aston-Jones and Cohen [1] proposed an adaptive gain theory of LC-NE function that suggests it plays an important role in modulating the trade-off between exploitation and exploration, which ultimately optimizes behavioral performance [1]. During the phasic LC mode, LC activity exhibits a strong phasic increase in response to task-relevant stimuli. Conversely, during tonic LC mode, LC experiences an increased level of baseline activity and absence of phasic responses [1], [4].

In order to further investigate the links between the LC-NE system and brain function in humans, recent studies have suggested using pupil diameter as an index of LC activity [5]. Although a direct anatomical connection between the LC and pupillary dilator muscle is yet to be determined, baseline pupil diameter has been found to closely track the dynamics of tonic LC activity in monkeys [6].

While simultaneous pupil and fMRI recording enables inferences about the relationship between LC activity and activity in spatially localized cortex [7], the limited temporal resolution of fMRI makes it difficult to infer timing relationships in neural processing. While its spatial resolution is limited, scalp electro-encephalography (EEG) provides millisecond-range temporal resolution to allow such temporally precise inferences. However, with one notable exception [8], there have been no studies that examine the relationship between LC activity (indexed by pupillary measures) and scalp EEG. The study of Murphy et al. [8] mainly evaluated the utility of two candidate psychophysiological markers of LC activity, namely EEG event-related potentials (ERPs) and pupil diameter. The results of this study aligned with the adaptive gain theory: it was found that prestimulus pupil diameter exhibited an inverted U-shaped relationship to P3 [8]. Absent this one study, the potential relationships between LC activity and neural processing at specific poststimulus times have remained largely unexplored.

While the study of Murphy et al. [8] was constrained to links between pupil diameter and two ERP components (the N1 and P3), our present study utilized a data-driven approach to learn the most task-relevant EEG projections spanning the entire trial, and examined the relationships between EEG fluctuations along these projections with pupil diameter. By exploiting this EEG single-trial variability, we were able to identify temporally specific task-relevant EEG components that are significantly linearly related with pupil diameter, uncovering key timing information for inferring connections between specific poststimulus neural processes and LC activity. We also investigated how prestimulus neural activity, specifically variability in the magnitude of alpha oscillations, correlates with poststimulus pupil dilation. EEG alpha power has been shown to negatively correlate with attention and subjects' task engagement prior to each trial [9]–[11]. Since the EEG single-trial variability provides information beyond what is reflected in behavioral response, in this way we provide further insight regarding the link between prestimulus and poststimulus cortical and subcortical processes underlying target detection.

## Methods

### Ethics Statement

This study was carried out in accordance with the guidelines and approval of the Columbia University Institutional Review Board. Written informed consents were obtained from all participants.

### Subjects and Behavioral Paradigm

Fifteen subjects (7 female; mean age 26.8, range  = 20–44 years) participated in the experiment. One participant was excluded because of excessive artifacts in the EEG data. All participants had normal or corrected-to-normal vision and no history of psychiatric illness or head injury.

An auditory oddball paradigm with 80% standard and 20% oddball (target) stimuli was used. This simple target detection task allowed subjects' minds to wander while maintaining near-perfect behavioral response accuracy. Standard stimuli were pure tones of frequency 350 Hz, while the target stimuli were broadband (laser gun) sounds. Stimuli were presented through speakers and each lasted for 200 ms with an inter-trial interval (ITI) sampled from a uniform distribution between 2 s and 3 s. Subjects were instructed to press a button on a gamepad with their right index finger as soon as they heard the target sound. For each subject, there were a total of 75 target and 300 standard trials (75 trials for each run, 5 runs in total).

### Simultaneous EEG and Pupil Data Acquisition

Experiments were performed in a dark electro-magnetically shielded room, and thus controlled for visual sensory input that might affect the pupil diameter. Throughout the entire experiment, subjects' pupil diameter was measured at a rate of 1 kHz with an EyeLink 1000 infrared eye-tracker (SR Research, Mississaugu, ON, Canada). Subjects were instructed to fixate on a central white cross for the duration of each run. Subjects' EEG was simultaneously recorded using a 64 scalp electrode ActiveTwo system (Biosemi, The Netherlands) with electrodes in the standard 10/20 configuration. EEG was recorded at a sampling rate of 2048 Hz.

### EEG and Pupil Data Pre-processing

For continuous EEG data, a 0.5 Hz high-pass filter was used to remove DC drift, and 60 Hz and 120 Hz notch filters were used to remove electrical line noise. The EEG data were then re-referenced to the average. An anti-alias filter was applied and the data were then down-sampled to 1 kHz to match the sampling rate of the pupil data. For continuous pupil diameter data, periods of blinks were detected using Eyelink's on-line parsing system, and then linearly interpolated in Matlab (The Mathworks, Natick, MA, USA). In order to compare within and across subjects, the pupil trace for each trial was normalized to the mean pupil diameter of the corresponding subject, resulting in a percentage pupil diameter change.

EEG and pupil diameter data were epoched identically, from 1 s prior to 2 s following each stimulus, with baseline removal on the last 500 ms prior to stimulus onset. Trials with either excessive noise in the EEG or pupillary data were manually identified and removed. Similarly, trials that resulted in behavioral error (i.e. missed targets or incorrectly responded to standards) were excluded from future analyses. We also rejected target trials whose preceding trial was also a target, since it was possible for the evoked pupil response of the first target trial to confound the pupil diameter baseline of the following trial.

### Single-trial EEG Analysis

We performed a single-trial analysis to discriminate the target trial EEG signal from the standard trial EEG signal using the sliding window logistic regression method of Parra et al. [12]–[14]. Here we present a brief overview.

The goal of this method is to find a projection of the multidimensional EEG signal, 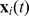, 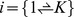 (*i* indexes trials) within a short time window that achieves maximal discrimination between standard and target trials. All time windows had a width of *M* = 50 ms and the window center, 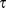, was shifted from 0 ms to 1000 ms relative to stimulus onset, in 25 ms increments. We used logistic regression to learn the 64-channel spatial weighting, 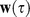, that maximally discriminated conditions, arriving at the projection,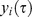, for each trial *i* and a given window 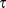.
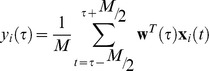



Note that we use the average projection in each temporal window, 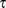, which tends to be less sensitive to noise [14]. Classifier performance for each subject was estimated at different window centers by computing the area under the receiver operating characteristic (ROC) curve, termed A_z_, using a leave-one-out cross validation procedure. By performing the leave-one-out test after randomly permuting the trial labels, and repeating this permutation method 100 times for each subject, we constructed a null distribution of A_z_ values across the subjects and determined the p<0.01 threshold.

For each window 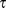 we also generated the forward model 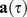,
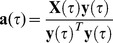
where we now organize 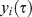 as a vector 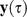, where each row is from trial *i*, and we organize 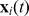 as a matrix, 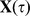, where rows are channels and columns are trials, all for time window 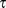. These forward models can be viewed as scalp plots and interpreted as the coupling between the discriminating components and the observed EEG.

### Pupil Diameter Analysis

We examined two pupil diameter measurements, namely the baseline pupil diameter and the evoked pupil dilation. The baseline pupil diameter was defined as pupil diameter at stimulus onset time. The evoked pupil dilation was defined as the maximum percentage deviation from baseline pupil diameter within each epoch.

### Envelope of Prestimulus Alpha Oscillations

Previous work has suggested a link between prestimulus oscillatory measures and poststimulus evoked responses, particularly in the alpha band [15]. Therefore apart from baseline pupil diameter, we also investigated the envelope of EEG alpha oscillations as another prestimulus measurement. Note that the envelope of the oscillation is related to its instantaneous power by a constant.

To estimate the envelope of prestimulus alpha oscillations for each target trial, we first performed an independent component analysis (ICA) on the EEG data. We selected a single “alpha component” based on two criteria: 1) the component with the highest ratio of mean power in the 8–12 Hz alpha band relative to mean power in adjacent bands (5–8 Hz theta band and 12–20 Hz beta band) which 2) also had a posterior scalp topography. We estimated the alpha activity from this component using a band-pass filter (with a bandwidth of 4 Hz) centered on the subject-specific alpha frequency, which was determined based on the peak in the power spectrum of the unfiltered EEG. A Hilbert transform [16] was used to construct the envelope of alpha oscillations across time. Lastly, the estimation of prestimulus alpha oscillations for each trial was obtained by averaging this envelope in the −500 to 0 ms time range prior to the stimulus.

### Generalized Linear Model Analysis and Statistics

**Figure 1:**
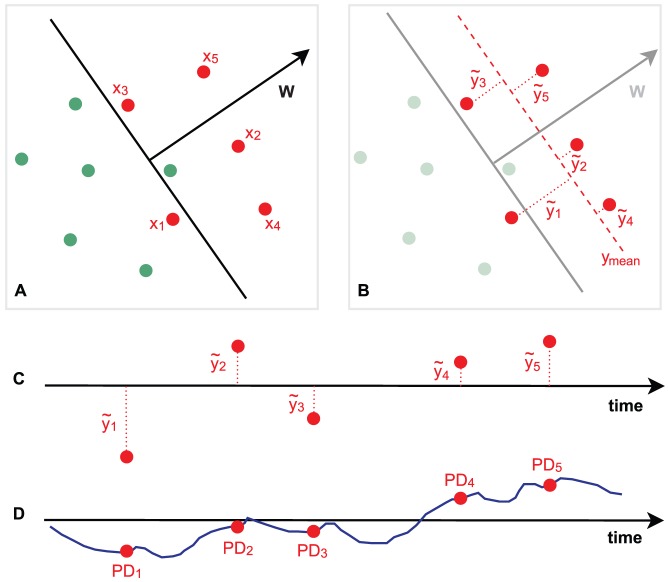
Methodology for correlating EEG single-trial variability with baseline pupil diameter. (A) We first estimate **w**, which is a linear weighting on the EEG sensors that maximally discriminates stimulus conditions: targets (red) vs. standards (green), shown in two dimensions for illustration purposes. This determines a task-relevant projection of the data, where the distance to the decision boundary reflects the decision certainty of the classifier. (B) From **w** we compute 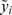, which is the demeaned classifier output, 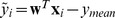, for each target trial, i. (In the text we refer to this variable as post-EEG_comp_). (C) Given the 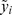
's (post-EEG_comp_) and their corresponding stimulus-onset time points, we compute the correlation with (D) baseline pupil diameter. This entire method was applied independently for multiple temporal windows, 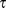, spanning the trial.

For each time window, 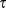, between 0 ms and 1000 ms poststimulus onset, we used a generalized linear model (GLM) to fit the de-meaned output, 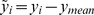, of the EEG discriminator for each trial, i (see Fig. 1), with the following four measurements that we described in the preceding sections. Note that for convenience we drop the 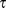 from our notation since a given 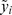 is always implicitly linked to a given time window 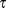
(i.e. the expressions 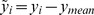 and 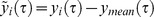 are equivalent, where 
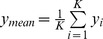
).

Before these analyses, all measurements were z-scored within each subject.

- reaction time (RT)
- magnitude of prestimulus alpha oscillations (pre-EEG_α_)
- baseline pupil diameter (pre-PD)
- evoked pupil dilation (post-PD)

We refer to the EEG discriminating component variability as post-EEG_comp_ (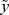). We performed independent hierarchical (single subject carried through to group level) GLM fits between each of the four measurements and 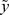
's spanning the entire trial. Each GLM was designed to examine both linear and quadratic relationships between measurements of interest, by including both linear and squared terms as regressors, and orthogonalizing the quadratic regressor to the linear regressor. For each GLM, we fit one of the four measurements to 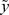. By repeating this fit for windows at different poststimulus times, we obtained a vector of coefficient estimates (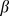) that showed a progression of linear and quadratic estimates over time. A large 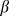 for the linear term is indicative of a strong linear relationship and a large 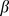 for the quadratic term indicates a strong quadratic relationship. Furthermore, the sign of the 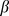 in the quadratic term indicates the direction of concavity/convexity: positive quadratic 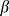 indicates that the relationship is U-shaped (convex), while negative quadratic 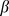 indicates an inverted U-shape (concave).

In order to estimate the significance level of these linear and quadratic relationships, as well as to correct for multiple comparisons, we applied threshold-free cluster enhancement (TFCE) [17]. The use of TFCE on the time series of GLM coefficient estimates ensured detection of both diffuse, low-amplitude correlations (i.e. weak but long-lasting) and sharp, local correlations (i.e. strong but short-lived). For each of the four measures we tested, we constructed null distributions of TFCE scores by permuting the vector of single-trial measurements 1000 times, computing the GLM fits, and applying TFCE. We used family-wise error (FWE) correction to determine the corrected p<0.05 threshold.

In order to tease apart correlates that were observable in RT from latent variability in the post-EEG_comp_, we repeated the GLM analyses after linearly regressing out RT from the discriminating component variability 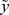.

Our primary analyses focused on linear and quadratic relationships between the EEG discriminating component variability of target trials and RT, pre-EEG_α_, pre-PD and post-PD. Additionally, we investigated pairwise linear and nonlinear relationships between these four attention-related measures through the previously described GLM methods. For pairwise quadratic GLM fits, the results and their interpretation depend on the form of the GLM, e.g. choosing which measurement is the predictor variable and which is the response variable. We assigned measurements to one of these variables based on our hypotheses regarding the LC adaptive gain theory. In particular, 1) we always fit pupil and EEG measures to RT since RT is a measure of task performance; 2) we always fit pupil measures to EEG measures since the latter better characterizes task performance than the former, and 3) we always fit prestimulus pupil measures to poststimulus pupil measures since the latter is an evoked response. In this way, we were able to study the relationships between behavioral, neural and pupillary measurements both before and after the stimulus.

## Results

### Behavioral Performance

All fourteen subjects performed the task at high accuracy with 99.3%±0.2% of targets correctly detected. Average reaction time was 394.4±29.1 ms.

### Trial-averaged Evoked Analysis

**Figure 2:**
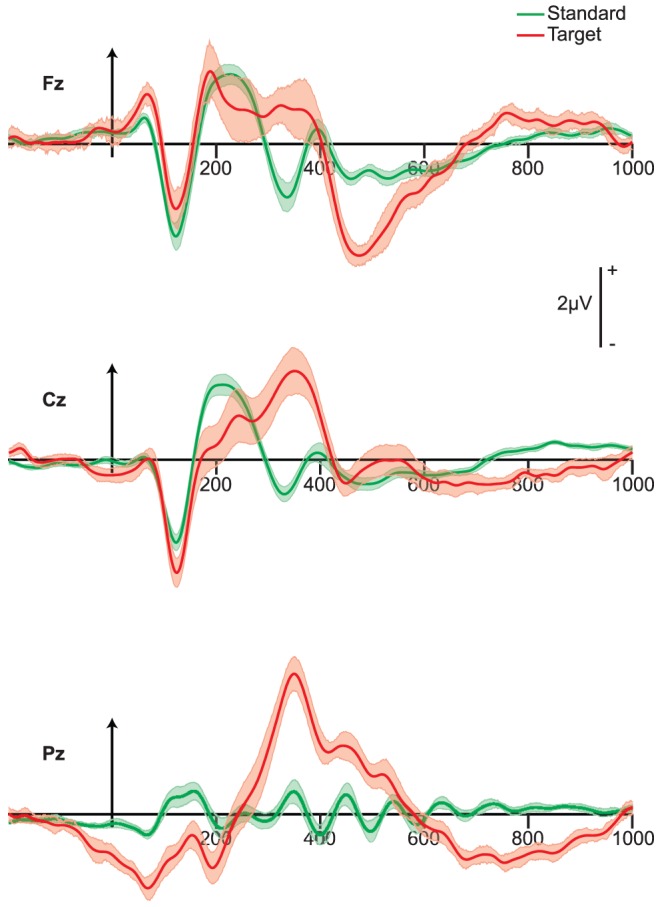
Event-related potentials at electrodes Fz, Cz and Pz. Shown are grand average (N = 14) stimulus-locked curves from 200 ms prestimulus to 1000 ms poststimulus for target (red) and standard (green) stimuli, with shaded bands indicating standard error.

We first examined trial-averaged event-related potentials (ERPs) and evoked pupil responses for both target and standard trials, quantifying the magnitude of the differences in the average evoked activity. Figure 2 shows the resulting ERPs. The N1-P2 complex can be seen at fronto-central electrode sites (Fz, Cz), followed by the N2 component, which was larger for targets than standards on posterior scalp sites (shown on Pz). This is consistent with results from many oddball paradigms [18]. Also, the P3 component was evident and most prominent on the parietal (Pz) electrode, peaking at approximately 350 ms poststimulus.

**Figure 3:**
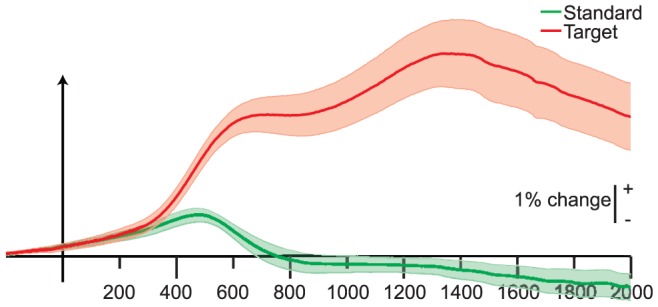
Evoked pupil dilation. Grand average (N = 14) stimulus-locked curves from 200 ms prestimulus to 2000 ms poststimulus for target (red) and standard (green) stimuli, with shaded bands indicating standard error. Traces have units of percentage change from the mean.

Both target and standard stimuli evoked pupil diameter increases (Fig. 3). An early dilation peak was seen at 500–600 ms poststimulus for both standard and target trials. This is consistent with the results of Steinhauer et al. [19], who described pupil dilations caused by inhibition of parasympathetic pathways. The primary dilation, i.e. the maximum pupil dilation evoked by target stimuli, was reached at approximately 1350 ms. Consistent with well-established pupillometry findings, the pupil dilation following target stimuli was larger than the dilation following standards [19].

### Single-trial Task-relevant EEG Components

**Figure 4:**
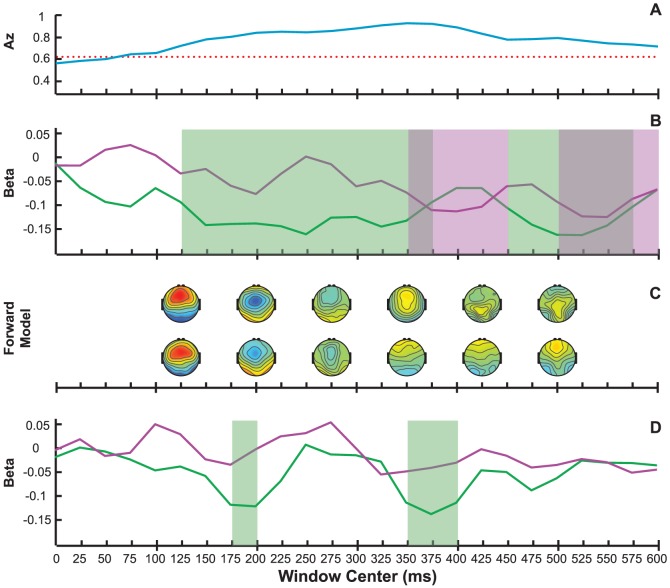
GLM fits of RT and baseline pupil diameter (pre-PD) to EEG components (post-EEG_comp_). Shown are group-level results for EEG windows spanning the trial, with shaded regions denoting p<0.05 (corrected) significance for linear (green) or quadratic (purple) relationships. (A) EEG classifier performance as defined by area under the ROC curve (blue trace). p = 0.01 (Az = 0.62) threshold is indicated with a red dotted line. (B) Linear (green) and quadratic (purple) GLM fit coefficient estimates, 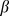, between RT and post-EEG_comp_ (classifier output *y*) as a function of window time. (C) Subset of scalp topographies generated using *y* (top row) and residual *y* (after regressing out RT, bottom row). (D) Linear (green) and quadratic (purple) GLM fit coefficient estimates between pre-PD and post-EEG_comp_.

We next looked at the single-trial EEG in order to relate fluctuations in pupil diameter with the temporally localized task-relevant EEG. Group mean single-trial EEG discriminator performance (shown in Fig. 4A) was significant for all consecutive windows between 75 ms and 850 ms poststimulus (p<0.01 for A_z_>0.621, computed via permutation test). The subject-averaged performance reached its peak of A_z_ = 0.92 at 350 ms. Corresponding forward models for a subset of windows with significant discrimination are shown in the top row of Figure 4C. Discriminating activity around 200 ms was strongest at central sites and more negative for targets compared to standards. This spatial distribution and peak latency was characteristic of the N2 component. In addition, strong positive activity at parietal sites lasted from 350 ms to 500 ms and was consistent with the P3.

### GLM Analysis

We next conducted our GLM analysis between poststimulus EEG discriminant component variability for target trials (which we refer to as post-EEG_comp_, representing 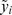
's for target trials for each window 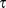) and behavioral and pupil diameter measurements. First we considered relationships between post-EEG_comp_ and RT. Figure 4B shows significant negative linear relationships between post-EEG_comp_ and RT for all but two consecutive windows from 125 ms to 575 ms, and significant negative quadratic relationships for all but one window inside the range of 350 ms to 600 ms. Previous work has shown that much of the LC activity and the late phases of the P3 response are locked to reaction time [1], [20]. In order to capture variability that is unique to the EEG latent states and not attributable to behavioral measures, such as RT, we regressed out RT from post-EEG_comp_. The bottom row in Figure 4C shows the resulting forward models after the linear contribution of RT variability was removed. The major difference between these scalp topologies and the ones prior to decorrelating with respect to RT was seen during the P3 time window of 350 ms to 500 ms. Regressing out RT significantly reduced the posterior activations typically associated with the P3b, while making more apparent the frontal contributions associated with the frontal P3 (P3f) (see Figure S1). These results are consistent with the findings of Gerson et al. [20]; they showed a fronto-parietal transition of the P3 complex that begins with a stimulus-locked P3f and ends with a classic response-locked parietal P3b topography.

Figure 4D shows the results of fitting linear and quadratic models of pre-PD to residual post-EEG_comp_ (after regressing out RT from post-EEG_comp_). We found only significant negative linear relationships between pre-PD and post-EEG_comp_, while the timing of the significant linear relationships aligned with the latencies of the N2 and P3 ERP components. Supporting Figure S2 shows the results of GLM fits between pre-PD with post-EEG_comp_ before and after regressing out RT from post-EEG_comp_. Though regressing out RT from post-EEG_comp_ did change the forward models (as shown in Figure 4C), it did not significantly change the statistical significance or effect size of the linear relationships between pre-PD and post-EEG_comp_ (compare the orange vs. green curves).

**Figure 5:**
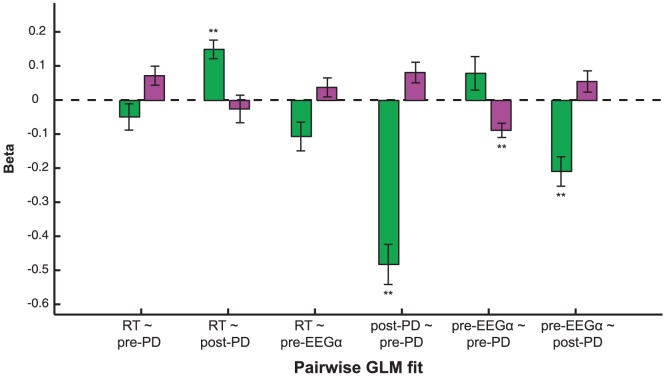
Pairwise GLM fits between RT, baseline pupil diameter, evoked pupil dilation and magnitude of prestimulus EEG alpha. Shown are linear (green) and quadratic (purple) GLM fit coefficient estimates (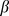) between the four measurements: RT, pre-PD, post-PD and pre-EEG_α_. For each pairwise GLM fit, measurement label on the top is the response variable, while measurement on the bottom is the predictor variable. Standard error bars are across subjects (N = 14). Significant linear or quadratic relationships (p<0.01, corrected) are denoted by double asterisks. Note that we obtained no results with 0.01<p<0.05.

We investigated several additional relationships in our analysis. First we looked at whether prestimulus alpha (pre-EEG_α_) covaried with post-EEG_comp_, and no significant relationships between this measurement and post-EEG_comp_ were found. We also investigated the relationship between post-PD and post-EEG_comp_ for both are poststimulus evoked response; neither linear nor quadratic relationships between these two measurements reached significance level (p<0.05) after multiple comparison correction. Figure 5 shows pairwise relationships between the four measurements. Notably, post-PD has a negative linear relationship with pre-EEG_α_. We also observed that large pupil dilations were linked to longer RTs.

## Discussion

**Figure 6:**
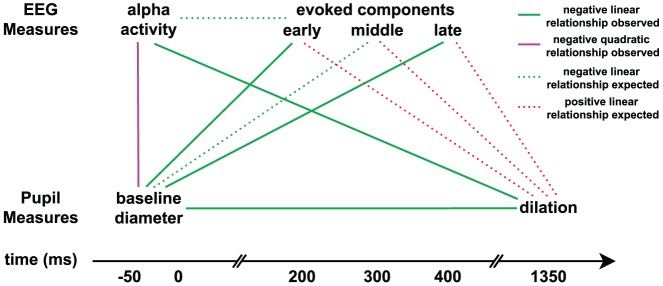
Summary of pairwise relationships between pupil and EEG measures. All measures are aligned according to their temporal order. Prestimulus measures of endogenous attentional state include baseline pupil diameter and magnitude of prestimulus EEG alpha oscillations. Poststimulus measures (evoked responses) include EEG discriminating components at different window offsets (early ∼200 ms, middle ∼300 ms, late ∼400 ms) and evoked pupil dilation. Significant (p<0.05, corrected) findings are shown with solid lines, where red indicates positive linear relationship, green indicates negative linear, and purple indicates negative quadratic. Dotted lines denote relationships that were hypothesized based on previous literature but not detected in our study.

Our study investigated relationships between prestimulus and poststimulus pupil and EEG measurements that have all been independently linked to attention, arousal, or anticipation. A summary of our findings is shown graphically in Figure 6. We based our analysis only on target trials (i.e. for identical stimuli), so the obtained EEG variability was driven purely by endogenous factors, primarily instantaneous attention to the task and anticipation for upcoming target stimuli. Our results enable us to make inferences about functional interactions between prestimulus and poststimulus neural processes that are known to modulate with various endogenous brain states.

Consistent with previous literature [20], we found that much of the poststimulus evoked EEG single-trial variability is reflected in the RT (Fig. 4B). This demonstrated the need to regress out the RT variability, since our goal was to study latent brain states. Within our Discussion section, unless otherwise noted, post-EEG_comp_ refers to the residual
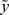
's of targets after regressing RT out of the classifier output.

### Baseline Pupil Diameter Correlates with Early and Late Neural Responses

Prior work using highly invasive neurophysiological recordings in primates has demonstrated a close link between prestimulus pupil diameter and tonic LC activity [1], which can be exploited to make inferences about LC activity in humans [8]. In contrast to the work of Murphy et al. [8], which investigated single-trial pupil diameter correlates with only N1 and P3 ERP components, our study utilized the most task-relevant EEG projections spanning the entire trial and investigated their pupil diameter correlates. This approach enabled us to temporally localize specific task-related EEG components that are significantly correlated with pupil measures, teasing them apart from EEG components that do not possess such pupil correlates.

We found negative linear relationships between post-EEG_comp_ and pre-PD within two poststimulus time ranges. The late post-EEG_comp_, at 350–400 ms, are closely related to the P3 ERP component, as evidenced by the ERP traces and forward models. Significant linear relationships between pre-PD and late post-EEG_comp_ suggest a connection between underlying tonic LC activity (indexing arousal) and decision-related processing. We found no significant linear relationships between middle post-EEG_comp_ and pre-PD, suggesting that tonic LC activity does not influence task-related neural processing around 300 ms poststimulus.

The early post-EEG_comp_ around 175–200 ms are representative of the N2 ERP component, which has not yet been studied in relation to pupil measurements. Our study found no linear relationships between pre-PD and neural responses at the typical N1 time, consistent with the findings of Murphy et al. [8]. Nevertheless, the linear relationship we found between pre-PD and EEG activity at 200 ms possibly indicates the influence of prestimulus tonic LC activity on early sensory processing of target stimuli. Recent work suggested a positive correlation between phasic LC-NE activity and N2 amplitude [21]. Here we found that EEG discriminating components in the N2 and P3 ranges both correlate with prestimulus pupil diameter. Along with previous studies, this suggests that EEG activity at the latency of the N2 might be closely connected with LC activity. Our finding is consistent with the EEG-fMRI study of Walz et al. [22], which found strong functional connections between an anterior cingulate cortex correlate of N2-range EEG variability and a midbrain correlate of P3-range EEG variability.

### Evoked Pupil Dilation Correlates with Prestimulus Pupil Diameter but Not Evoked EEG Responses

As described by Aston-Jones and Cohen [1], the fluctuations of tonic LC activity are known to affect the stimulus-driven phasic responses. In this study, we found that maximum pupil dilation is negatively linearly correlated with baseline pupil diameter, which is consistent with the reports of Murphy et al. [8] and de Gee et al. [23]. We also observed negative linear relationships between post-PD and pre-EEG_α_; further discussion regarding this relationship can be found below.

Since phasic LC activity has been linked to both decision-related pupil dilations and the P3 ERP component, we initially designed this study expecting to observe a relationship between our post-PD measurement and post-EEG_comp_. However, although we found both post-PD and post-EEG_comp_ to be significantly linearly correlated with pre-PD, our analysis did not detect any linear or quadratic correlations between these two measures (refer to dotted red lines in Fig. 6). Recent work also failed to detect such expected correlations between P3 ERP component variability and pupil dilations [8].

Murphy et al. [8] discussed possible sources of variance that obscured a more direct relationship between pupil dilation and the P3 ERP component. One of the measurement issues mentioned is that pupil dilation and P3 have markedly contrasting latencies, and it is possible that they may reflect different combinations of distinct information processing stages. Multimodal studies with simultaneous pupillometry, EEG, and fMRI recording may be required for better understanding why this correlation is not seen with EEG and pupil measures alone.

As discussed in the previous section, decision-related cognitive processing may act as an early (prior to the P3) mediating factor in a chain of processes, while internally-driven performance monitoring after the behavioral response likely acts as a later mediator of neural activity. Furthermore, as Murphy et al. pointed out [8], the pupil dilation occurs on a much longer time scale than evoked neural responses, which allows additional endogenous processes to participate and influence the response. These mediating factors can potentially add enough variance to obscure a more direct connection between pupil dilation and neural responses.

Very recent work using pupillometry and a difficult visual discrimination task [23] suggested that LC phasic activity is not based solely on the outcome of decision processes, but might instead be linked to accumulation of sensory evidence and intrinsic biases that lead up to and influence the decision. This is in contrast to the previous model of the LC phasic response being driven by the termination of decision-related processes [24]. It is unknown whether the literature's discrepant conclusions are due to differences across tasks, species, individuals, or a functional dissociation between the LC and pupil size. Future studies should investigate these ideas using simultaneous EEG-pupillometry with difficult or protracted decision tasks.

### RT is Not Closely Linked with Baseline Pupil Diameter

Previous animal studies have reported an inverted U-shaped relationship between tonic LC activity and task performance [1], in which task performance is optimal during intermediate states of tonic LC activity. While Murphy et al. [8] found no relationship between prestimulus pupil diameter and RT, they reported an inverted U-shaped trend between prestimulus pupil diameter and RT coefficient of variation (CV), and thus suggested that this quadratic relationship between pupil diameter and task performance supports the hypothesis that prestimulus pupil diameter is a valid proxy for tonic LC activity in humans. A similar analysis on our data did not show such an inverted U-shape relationship. There are several possible explanations for this. However, given our focus was on relating trial-to-trial variability in pupillary and EEG measures, and RT CV is by definition a measure that requires binning/aggregating trials, we choose not to speculate on reasons for our lack of this corroborative finding.

Our study found no significant relationships between RT and pre-PD. One possible explanation is that while baseline pupil diameter fluctuates from trial to trial, RT does not exhibit much variability since all subjects responded quickly and achieved near-perfect accuracy throughout the experiment. This lack of correlation between prestimulus pupil diameter and RT suggests that tonic LC activity is not likely the source of RT variability in our data.

Although we found no linear relationship between pre-PD and RT, we observed both negative linear and quadratic relationships between RT and post-EEG_comp_ for the majority of windows spanning the trial, which was expected based on previous findings of negative correlations between RT and ERP amplitudes [25].

### Prestimulus Alpha Modulations Correlate with Evoked Pupil Dilation

It is well known that power in the EEG alpha band (8-12 Hz) is negatively correlated with attention to the task [26]. High prestimulus alpha (i.e., low attention) has been linked with decreased behavioral response accuracy, but not with longer RTs [15]. Consistent with previous literature, we found no linear relationship between prestimulus alpha and RT, but we did find a significant negative linear relationship between the magnitude of prestimulus alpha oscillations and the evoked pupil dilation. To the best of our knowledge, pupil measures have not been studied in relation to EEG alpha until now. Since pupil dilation has been proposed to index phasic LC activations [8], [23], our observed negative linear relationship between post-PD and pre-EEG_α_ could represent a connection between prestimulus attentional state and poststimulus phasic LC activation.

Since we found evoked pupil dilations to have strong negative linear relationships with both pre-EEG_α_ and pre-PD, we might expect to find a linear relationship between these two measures of prestimulus attentional states. However, we found a negative quadratic relationship between pre-PD and pre-EEG_α_. One possible interpretation of this finding is that it represents an exogenous shifting of auditory attention, observable via the alpha activity in occipital cortex, which is the largest observable source of alpha in scalp EEG. Since alpha activity in auditory cortex is not observable in scalp EEG, we cannot directly confirm this interpretation. However indirect push-pull of alpha activity between auditory attention and visual attention has been previously observed in oddball tasks in which attention was exogenously shifted, via a cue, between auditory and visual stimuli [27].

### Conclusions

In this study, we used a data-driven analysis to investigate the complex dynamic relationships between pupillary measures and EEG variability. Specifically, we investigated both linear and quadratic relationships between prestimulus (baseline pupil diameter, magnitude of EEG alpha oscillations) and poststimulus (evoked pupil dilation, temporally-specific EEG components) physiological variables that have all been independently linked to attention. Here by identifying correlates of temporally specific post-EEG_comp_ with pre-PD, we provide key timing information regarding functional relationships between specific poststimulus endogenous processes and prestimulus pupil diameter, which is thought to index tonic LC activity. We believe our findings will promote future studies that utilize noninvasive measurements to further investigate and index LC activity in a more direct way.

## Supporting Information

Figure S1
**Statistical differences between forward models before and after regressing out RT.** Pairwise t-tests were conducted on each electrode using forward models before and after regressing out RT, with 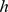 denoting the test decision (0 or 1) and 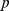 denoting the significance level. Shown are 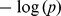 and 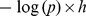 values (top and bottom row, respectively) for selected window.(DOCX)

Figure S2 **GLM fits of baseline pupil diameter (pre-PD) to EEG components (post-EEG_comp_).** Shown are group-level linear relationships between pre-PD and post-EEG_comp_ before (orange trace) and after (green trace) regressing out RT. Shaded regions denote p<0.05 (corrected) significance. These significant regions did not change after regressing out RT.
(DOCX)
