# Supplementary material for: Your Eyes Give You Away: Prestimulus Changes in Pupil Diameter Correlate with Poststimulus Task-Related EEG Dynamics
Source: PLoS One. 2014 Mar 11;9(3):e91321. doi: 10.1371/journal.pone.0091321 (PMC3950210; doi:10.1371/journal.pone.0091321)
Supplement: Figure S1 — Statistical differences between forward models before and after regressing out RT. Pairwise t-tests were conducted on each electrode using forward models before and after regressing out RT, with denoting the test decision (0 or 1) and denoting the significance level. Shown are and values (top and bottom row, respectively) for selected window. (DOCX) [file pone.0091321.s001.docx]

SUPPLEMENTARY MATERIAL

Your Eyes Give You Away: Prestimulus Changes in Pupil Diameter Correlate with Poststimulus Task-related EEG Dynamics

Linbi Hong, Jennifer M. Walz and Paul Sajda


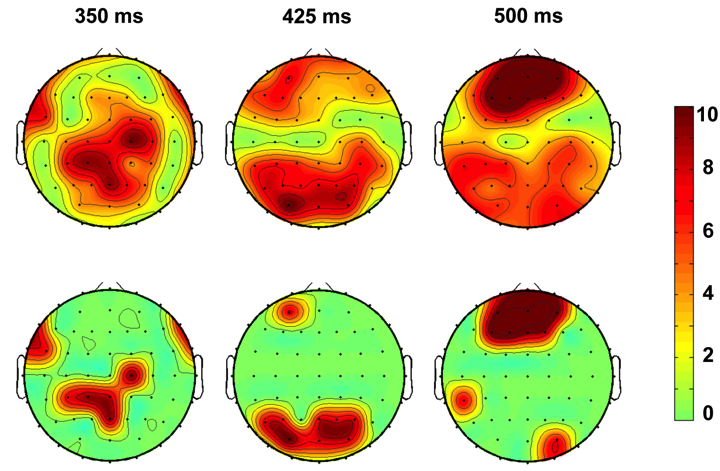


**Figure S1.** Statistical differences between forward models before and after regressing out RT. Pairwise t-tests were conducted on each electrode using forward models before and after regressing out RT, with $h$ denoting the test decision (0 or 1) and $p$ denoting the significance level. Shown are $-\log(p)$ and $-\log(p)\times h$ values (top and bottom row, respectively) for selected window.
