# Supplementary material for: Your Eyes Give You Away: Prestimulus Changes in Pupil Diameter Correlate with Poststimulus Task-Related EEG Dynamics
Source: PLoS One. 2014 Mar 11;9(3):e91321. doi: 10.1371/journal.pone.0091321 (PMC3950210; doi:10.1371/journal.pone.0091321)
Supplement: Figure S2 — GLM fits of baseline pupil diameter (pre-PD) to EEG components (post-EEGcomp). Shown are group-level linear relationships between pre-PD and post-EEGcomp before (orange trace) and after (green trace) regressing out RT. Shaded regions denote p<0.05 (corrected) significance. These significant regions did not change after regressing out RT. (DOCX) [file pone.0091321.s002.docx]

SUPPLEMENTARY MATERIAL

Your Eyes Give You Away: Prestimulus Changes in Pupil Diameter Correlate with Poststimulus Task-related EEG Dynamics

Linbi Hong, Jennifer M. Walz and Paul Sajda


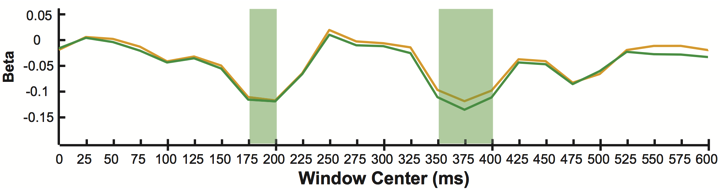


**Figure S2. GLM fits of baseline pupil diameter (pre-PD) to EEG components (post-EEG_comp_).** Shown are group-level linear relationships between pre-PD and post-EEG_comp_ before (orange trace) and after (green trace) regressing out RT. Shaded regions denote p < 0.05 (corrected) significance. These significant regions did not change after regressing out RT.
